# *S*-5-Amino-2-(dimethyl­ammonio)phenyl sulfothio­ate

**DOI:** 10.1107/S1600536809016912

**Published:** 2009-05-14

**Authors:** Gordana Pavlović, Livio Racané, Vesna Tralić-Kulenović

**Affiliations:** a Faculty of Textile Technology, Laboratory of Applied Chemistry, University of Zagreb, Prilaz baruna Filipovića 28a, HR-10000 Zagreb, Croatia

## Abstract

The title compound, C_8_H_12_N_2_O_3_S_2_, has been isolated as a by-product in the synthesis of methyl­ene blue dye. The compound crystallizes with four independent mol­ecules in the unit cell (*Z*′= 4). The zwitterionic form of the mol­ecule was established on the basis of the hydrogen atom located at the dimethyl­amino group. The crystal structure is dominated by inter­molecular hydrogen bonds of the N—H⋯O type formed between amino and ammonio N—H groups and O atoms from the sulfothio­ate group. There are in addition two weak inter­molecular N—H⋯N inter­actions and some non-conventional C—H⋯O hydrogen bonds.

## Related literature

For the preparation, see: Bennett & Bell (1943 ▶); Bogert & Updike (1927 ▶); Leventis *et al.* (1997 ▶). For information on methyl­ene blue see: Hunger (2003 ▶); Zollinger (1991 ▶). For bond-length data, see: Allen *et al.* (1987 ▶); Bertolasi *et al.* (1999 ▶); Trinajstić (1968 ▶).
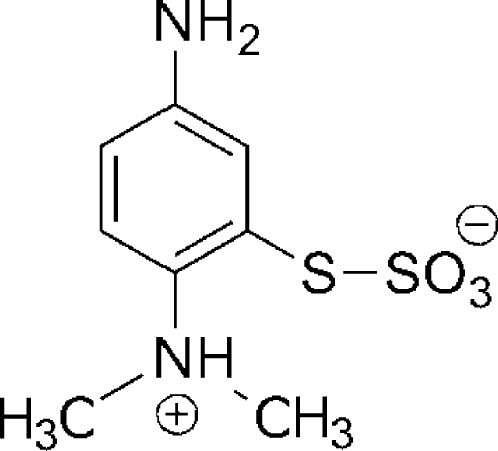


## Experimental

### 

#### Crystal data

C_8_H_12_N_2_O_3_S_2_
*M* *_r_* = 248.32
Triclinic, 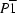

*a* = 10.4173 (2) Å
*b* = 14.1160 (4) Å
*c* = 15.3048 (4) Å
α = 93.474 (2)°
β = 101.0918 (19)°
γ = 93.0199 (19)°
*V* = 2199.73 (10) Å^3^
*Z* = 8
Mo *K*α radiation
μ = 0.47 mm^−1^
*T* = 296 K
0.67 × 0.44 × 0.28 mm

#### Data collection

Oxford Diffraction Xcalibur diffractometer with Sapphire 3 CCD detector
Absorption correction: multi-scan *CrysAlis RED* (Oxford Diffraction, 2006 ▶). *T*_min_ = 0.66, *T*_max_ = 0.88
39152 measured reflections
9530 independent reflections
6061 reflections with *I* > 2σ(*I*)
*R*_int_ = 0.029

#### Refinement

*R*[*F* ^2^ > 2σ(*F* ^2^)] = 0.047
*wR*(*F* ^2^) = 0.131
*S* = 0.96
9530 reflections
585 parameters
2 restraints
H atoms treated by a mixture of independent and constrained refinement
Δρ_max_ = 0.97 e Å^−3^
Δρ_min_ = −0.34 e Å^−3^

### 

Data collection: *CrysAlis CCD* (Oxford Diffraction, 2006 ▶); cell refinement: *CrysAlis RED* (Oxford Diffraction, 2006 ▶); data reduction: *CrysAlis RED*; program(s) used to solve structure: *SHELXL97* (Sheldrick, 2008 ▶); program(s) used to refine structure: *SHELXL97* (Sheldrick, 2008 ▶); molecular graphics: *ORTEP-3 for Windows* (Farrugia, 1997 ▶) and *Mercury* (Macrae *et al.*, 2008 ▶); software used to prepare material for publication: *SHELXL97*.

## Supplementary Material

Crystal structure: contains datablocks I, global. DOI: 10.1107/S1600536809016912/bg2255sup1.cif

Structure factors: contains datablocks I. DOI: 10.1107/S1600536809016912/bg2255Isup2.hkl

Additional supplementary materials:  crystallographic information; 3D view; checkCIF report

## Figures and Tables

**Table 1:** Hydrogen-bond geometry (Å, °)

*D*—H⋯*A*	*D*—H	H⋯*A*	*D*⋯*A*	*D*—H⋯*A*
N1*A*—H1*AA*⋯O2*A*^i^	0.86 (3)	2.40 (3)	3.204 (3)	157 (3)
N1*A*—H1*AB*⋯O3*B*^i^	0.89 (3)	2.38 (3)	3.189 (3)	152 (3)
N2*A*—H2*A*⋯O3*A*	0.81 (3)	2.31 (2)	2.983 (3)	141 (2)
N2*A*—H2*A*⋯O3*B*^ii^	0.81 (2)	2.48 (2)	3.003 (3)	124 (3)
N1*B*—H1*B*⋯O1*B*	0.86 (2)	2.28 (3)	2.940 (3)	134 (2)
N1*B*—H1*B*⋯O1*A*^ii^	0.86 (2)	2.45 (2)	3.016 (3)	124 (1)
N2*B*—H2*BA*⋯O2*B*^iii^	0.86 (2)	2.48 (2)	3.266 (3)	153 (3)
N2*B*—H2*BB*⋯O1*A*^i^	0.87 (3)	2.39 (3)	3.237 (3)	163 (3)
N1*C*—H1*CA*⋯O2*C*^iv^	0.81 (3)	2.44 (3)	3.149 (3)	147 (3)
N1*C*—H1*CB*⋯O3*D*^v^	0.83 (3)	2.55 (3)	3.349 (3)	162 (3)
N2*C*—H2*C*⋯O2*C*	0.82 (3)	2.41 (3)	2.982 (3)	128 (3)
N2*C*—H2*C*⋯N2*D*^vi^	0.82 (2)	2.44 (3)	3.122 (4)	143 (3)
N1*D*—H1*D*⋯N1*C*^vii^	0.81 (3)	2.31 (3)	3.013 (3)	146 (3)
N2*D*—H2*DA*⋯O1*D*^viii^	0.79 (3)	2.30 (3)	3.040 (4)	155 (3)
N2*D*—H2*DB*⋯O3*C*^ix^	0.99 (3)	2.40 (3)	3.314 (4)	154 (2)
C8*A*—H8*AA*⋯O2*D*^i^	0.96	2.37	3.273 (3)	157
C8*B*—H8*BA*⋯O3*D*^i^	0.96	2.54	3.437 (4)	155
C7*D*—H7*DA*⋯O2*B*^x^	0.96	2.51	3.370 (4)	149

Symmetry codes: (i) 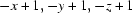; (ii) 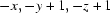; (iii) 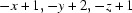; (iv) 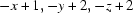; (v) 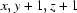; (vi) 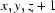; (vii) 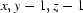; (viii) 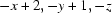; (ix) 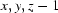; (x) 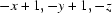.

**Fig. 1.**
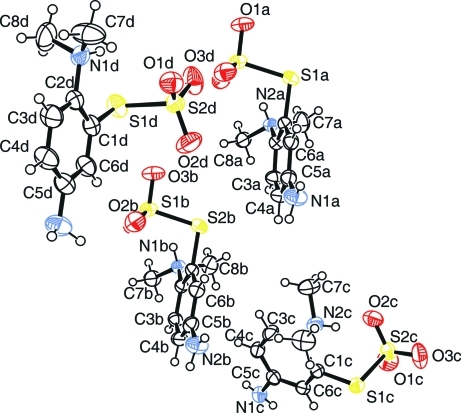
The molecular structure of (I) with the atom numbering scheme. Displacement ellipsoids are drawn at the 50% probability level. Each molecule is denoted by letters A, B, C and D.

**Fig. 2.**
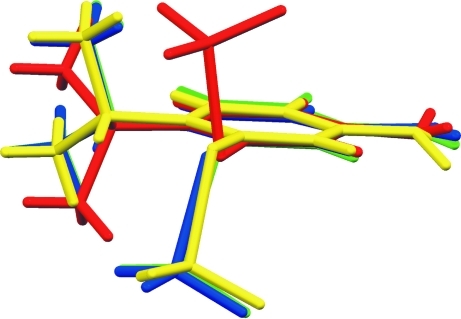
Molecular overlap of the four crystallographically independent molecules A, B, C and D in (I) (molecule A shown in green, B in blue, C in red and D in yellow).

**Fig. 3.**
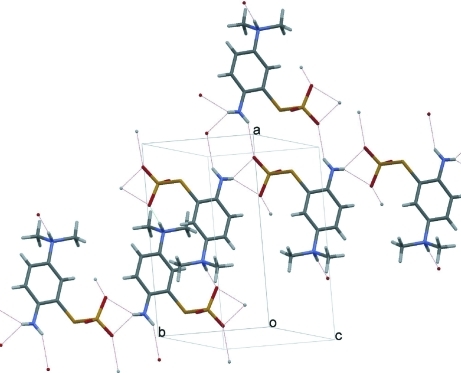
Crystal structure of (I).

## supplementary crystallographic information

### Comment

Phenothiazine dyes, from which methylene blue is the best known (Zollinger, 1991; Hunger, 2003) are a class of colorants with application in various fields. Methylene blue is commercially produced by oxidation of 4-*N*,*N*-dimethylaminoaniline with Na_2_Cr_2_O_7_ in the presence of Na_2_S_2_O_3_, followed by further oxidation in the presence of *N*,*N*-dimethylaniline, usually without isolation of intermediate 4-*N*,*N*-dimethylaminoaniline-2-tiosulfuric acid (Leventis *et al.*, 1997).

Following one of the well known methods for preparation of 4-*N*,*N*-dimethylaminoaniline-2-tiosulfuric acid (Bogert & Updike, 1927), we isolated *S*-5-amino-2-(dimethylammonio)phenyl sulfothioate (I) (Scheme 1), in zwitterionic form as a by-product. The product crystallizes with 4 independent molecules in the asymmetric unit, labelled as A, B, C and D (Fig.1.).

The molecule contains three substituents on the phenyl core (Fig.1.): the amino group, the dimethylammonium cation and the sulfothioate anion with individual geometries in accordance with literature data (Allen *et al.*, 1987).

The S—C bonds span the range 1.769 (2)  - 1.777 (2) Å reflecting aproximately 20% of π bond character (Trinajstić, 1968). The C_ar_—N bonds formed by amino groups have significant π character (1.367 (3), 1.363 (3), 1.379 (3),1.392 (4)Å in A, B, C and D respectively). Finally, C—N bonds in the *N*,*N*-dimethylammono moieties are essentially single bonds, with a 1.468 (3) - 1.500 (4) Å span. The C—S—S and O—S—S angles span the range 99.12 (8) - 100.37 (8)° and 100.32 (9) - 107.73 (9)°, respectively.

A molecular overlap of all four units (Fig.2.) indicates that the largest conformational difference between them arises in the spatial orientation of the dimethylammonio units relative to the phenyl rings, as well as in the sulfothioate part of the molecule While the conformations of molecules A (in green in fig. 2) and B (blue) are almost identical, molecule D (yellow) exhibits some conformational differences and molecule C (red) has a completely different spatial orientation of the mentioned substitutents. (See the Supplementary Material for torsion angles defining their geometries)

The rather complex hydrogen bonding network includes three fairly strong N—H···O intramolecular H-bonds and a number of N—H···O, N—H···N and a few non-conventional C—H···O intermolecular H-bonds (Fig. 3 and Table 1). All amino as well as ammonio NH's participate in N—H···O H-bond formation, with almost all nitrogens acting as double proton donors and many oxygens as double proton acceptors (Table 1). There are a couple of homonuclear N—H···N intermolecular interactions with N···N values in the range 3.013 (3)—3.122 (4) Å which compares fairly well with the mean value N···N = 2.97 (10) Å found by Bertolasi and co-workers for non-resonant N—H···N intermolecular hydrogen bonds in pyrazoles (Bertolasi *et al.*,1999). Finally, there are some non-conventional C—H···O bonds linking Car-H groups and S—SO_3_^-^ fragments (Table 1, three final entries).

### Experimental

*N*,*N*-dimethylaniline was dissolved in aqueous HCl and nitrosilated with NaNO_2_ (Bennett & Bell, 1943). The resulting crude 4-nitroso-*N*,*N*-dimethylaniline hydrochloride was isolated and dissolved in aqueous acetic acid. The cold water solution of Na_2_S_2_O_3_ was added and the reaction mixture was stirred at 273 - 278 K for several hours (Bogert & Updike, 1927), and left for two days at room temperature. The crude product was filtered off, and crystallized from water. The *S*-2-amino-5-(dimethylammonio)phenyl sulfothioate has been isolated, but after standing of mother liquor in refrigerator for several weeks *S*-5-amino-2-(dimethylammonio)phenyl sulfothioate (1 b) has been crystallized in the form of gray-greenish prism, as well. Spectroscopic analysis, IR (ATR, cm^-1^): 3455 (w), 3403 (w), 3360 (w), 3328 (w), 3079 (w), 1631 (*m*), 1600 (*m*), 1496 (*m*), 1465 (*m*), 1369 (w), 1296 (w), 1211 (*s*), 1228 (*m*), 1049 (*m*), 1012 (*s*), 985 (*m*), 893 (w), 870 (*m*), 822 (*s*), 613 (*s*), 494 (*s*). ^1^H NMR (600 MHz, DMSO-d_6_):δ 9.62 (br s, 1H), 7.55 (d, 1H, J = 8.8 Hz), 6.96 (s, 1H), 6.75 (d, 1H, J = 8.8 Hz), 5.85 (br s, 2H), 3.13 (s, 6H). Analysis, calculated for C_8_H_12_N_2_O_3_S_2_: C 38.69, H 4.87, N 11.28%; found: C 38.42, H 4.96, N 11.13%.

### Refinement

Hydrogen atoms bonded to amino and ammonio nitrogens were found in the difference Fourier electron-density maps and freely refined . The exceptions were the H's attached to N2B which N-H distances wouldn't refie properly and were accordingly restrained to the target value of 0.87 (1) Å. In all cases *U*_iso_ (H) = 1.2 *U*_eq_ (N). All hydrogens attached to carbon atoms were located at calculated positions and refined by applying the riding model (*U*_iso_ (H) = 1.2 *U*_eq_ (C) and Csp2-H distance 0.93 Å; Csp3-H 0.96 Å and *U*_iso_ (H) = 1.5 *U*_eq_ (C).

### Crystal data

**Table:**

C_8_H_12_N_2_O_3_S_2_	*Z* = 8
*M**_r_* = 248.32	*F*(000) = 1040
Triclinic, *P*1	*D*_x_ = 1.500 Mg m^−^^3^
Hall symbol: -P 1	Mo *K*α radiation, λ = 0.71073 Å
*a* = 10.4173 (2) Å	Cell parameters from 16121 reflections
*b* = 14.1160 (4) Å	θ = 4.1–34.9°
*c* = 15.3048 (4) Å	µ = 0.47 mm^−^^1^
α = 93.474 (2)°	*T* = 296 K
β = 101.0918 (19)°	Prism, green
γ = 93.0199 (19)°	0.67 × 0.44 × 0.28 mm
*V* = 2199.73 (10) Å^3^	

### Data collection

**Table:**

Enhance (Mo) X-ray Source diffractometer	9530 independent reflections
Radiation source: fine-focus sealed tube	6061 reflections with *I* > 2σ(*I*)
graphite	*R*_int_ = 0.029
ω–scan	θ_max_ = 27.0°, θ_min_ = 4.1°
Absorption correction: multi-scan *CrysAlis RED* (Oxford Diffraction, 2006).	*h* = −13→13
*T*_min_ = 0.66, *T*_max_ = 0.88	*k* = −18→18
39152 measured reflections	*l* = −19→19

### Refinement

**Table:**

Refinement on *F*^2^	Primary atom site location: structure-invariant direct methods
Least-squares matrix: full	Secondary atom site location: difference Fourier map
*R*[*F*^2^ > 2σ(*F*^2^)] = 0.047	Hydrogen site location: inferred from neighbouring sites
*wR*(*F*^2^) = 0.131	H atoms treated by a mixture of independent and constrained refinement
*S* = 0.96	*w* = 1/[σ^2^(*F*_o_^2^) + (0.0861*P*)^2^] where *P* = (*F*_o_^2^ + 2*F*_c_^2^)/3
9530 reflections	(Δ/σ)_max_ = 0.001
585 parameters	Δρ_max_ = 0.97 e Å^−^^3^
2 restraints	Δρ_min_ = −0.33 e Å^−^^3^

### Fractional atomic coordinates and isotropic or equivalent isotropic displacement parameters (Å^2^)

**Table:**

	*x*	*y*	*z*	*U*_iso_*/*U*_eq_	
S1A	0.27663 (7)	0.24900 (4)	0.55734 (4)	0.04401 (17)	
S2A	0.17068 (6)	0.27335 (4)	0.42951 (4)	0.03945 (16)	
S1B	0.17849 (6)	0.77005 (4)	0.44199 (4)	0.04073 (16)	
S2B	0.28071 (7)	0.74990 (4)	0.57259 (4)	0.04367 (17)	
S1C	0.90558 (6)	0.91691 (4)	1.04578 (4)	0.04246 (17)	
S2C	0.81879 (6)	0.81735 (5)	1.11966 (4)	0.04335 (17)	
S1D	0.61090 (7)	0.40268 (5)	0.03857 (5)	0.05126 (19)	
S2D	0.74027 (6)	0.32095 (5)	0.12351 (4)	0.04467 (17)	
O1A	0.10076 (19)	0.18205 (13)	0.40364 (13)	0.0593 (5)	
O2A	0.2658 (2)	0.29550 (14)	0.37611 (12)	0.0605 (5)	
O3A	0.09201 (19)	0.35090 (14)	0.44440 (13)	0.0616 (5)	
O1B	0.09958 (19)	0.84839 (14)	0.45331 (13)	0.0618 (5)	
O2B	0.2759 (2)	0.78960 (15)	0.38979 (13)	0.0622 (5)	
O3B	0.1079 (2)	0.67865 (13)	0.41843 (14)	0.0637 (6)	
O1C	0.7534 (2)	0.86953 (15)	1.17901 (13)	0.0648 (6)	
O2C	0.72798 (19)	0.75774 (13)	1.05336 (13)	0.0568 (5)	
O3C	0.9318 (2)	0.77085 (16)	1.15941 (15)	0.0755 (7)	
O1D	0.79910 (18)	0.26208 (13)	0.06400 (13)	0.0570 (5)	
O2D	0.8338 (2)	0.38584 (16)	0.18086 (13)	0.0723 (6)	
O3D	0.64858 (19)	0.27025 (16)	0.16585 (14)	0.0708 (6)	
N1A	0.6882 (2)	0.4774 (2)	0.60426 (19)	0.0580 (7)	
H1AA	0.724 (3)	0.534 (2)	0.611 (2)	0.070*	
H1AB	0.730 (3)	0.434 (2)	0.578 (2)	0.070*	
N2A	0.16030 (19)	0.42056 (14)	0.63661 (13)	0.0346 (4)	
H2A	0.123 (3)	0.3825 (18)	0.5971 (17)	0.042*	
N1B	0.16318 (18)	0.92695 (14)	0.64003 (13)	0.0342 (4)	
H1B	0.125 (2)	0.8810 (18)	0.6033 (17)	0.041*	
N2B	0.6913 (2)	0.97876 (18)	0.60693 (18)	0.0574 (6)	
H2BA	0.728 (3)	1.0353 (11)	0.611 (2)	0.069*	
H2BB	0.740 (3)	0.9344 (16)	0.592 (2)	0.069*	
N1C	0.5436 (2)	1.14941 (16)	0.96753 (16)	0.0456 (5)	
H1CA	0.468 (3)	1.155 (2)	0.9449 (19)	0.055*	
H1CB	0.567 (3)	1.167 (2)	1.021 (2)	0.055*	
N2C	0.7612 (2)	0.80939 (15)	0.87260 (14)	0.0408 (5)	
H2C	0.802 (3)	0.7874 (19)	0.9172 (19)	0.049*	
N1D	0.6621 (2)	0.29835 (17)	−0.13159 (15)	0.0514 (6)	
H1D	0.641 (3)	0.277 (2)	−0.088 (2)	0.062*	
N2D	0.9370 (3)	0.65704 (18)	−0.0356 (2)	0.0590 (7)	
H2DA	0.998 (3)	0.669 (2)	−0.058 (2)	0.071*	
H2DB	0.956 (3)	0.676 (2)	0.029 (2)	0.071*	
C1A	0.3592 (2)	0.36262 (15)	0.58949 (14)	0.0329 (5)	
C2A	0.2975 (2)	0.43572 (15)	0.62711 (14)	0.0335 (5)	
C3A	0.3675 (2)	0.52188 (17)	0.65689 (16)	0.0410 (6)	
H3A	0.3273	0.5706	0.6828	0.049*	
C4A	0.4955 (2)	0.53522 (17)	0.64817 (17)	0.0438 (6)	
H4A	0.5410	0.5934	0.6680	0.053*	
C5A	0.5594 (2)	0.46343 (17)	0.61024 (16)	0.0395 (5)	
C6A	0.4886 (2)	0.37750 (17)	0.58151 (15)	0.0383 (5)	
H6A	0.5292	0.3287	0.5562	0.046*	
C7A	0.1503 (3)	0.3849 (2)	0.72493 (18)	0.0560 (7)	
H7AA	0.1939	0.3268	0.7321	0.084*	
H7AB	0.0597	0.3733	0.7279	0.084*	
H7AC	0.1909	0.4316	0.7716	0.084*	
C8A	0.0830 (2)	0.50585 (17)	0.61936 (17)	0.0429 (6)	
H8AA	0.1103	0.5533	0.6680	0.064*	
H8AB	−0.0085	0.4880	0.6140	0.064*	
H8AC	0.0976	0.5311	0.5650	0.064*	
C1B	0.3640 (2)	0.86418 (15)	0.59945 (14)	0.0328 (5)	
C2B	0.2993 (2)	0.94133 (15)	0.62723 (14)	0.0314 (5)	
C3B	0.3651 (2)	1.03014 (16)	0.64587 (15)	0.0351 (5)	
H3B	0.3223	1.0816	0.6645	0.042*	
C4B	0.4937 (2)	1.04259 (17)	0.63691 (16)	0.0397 (6)	
H4B	0.5362	1.1030	0.6483	0.048*	
C5B	0.5620 (2)	0.96619 (18)	0.61100 (15)	0.0389 (5)	
C6B	0.4939 (2)	0.87747 (17)	0.59219 (16)	0.0389 (5)	
H6B	0.5369	0.8258	0.5743	0.047*	
C7B	0.0834 (2)	1.01044 (18)	0.61966 (19)	0.0477 (6)	
H7BA	0.1103	1.0601	0.6663	0.072*	
H7BB	−0.0076	0.9918	0.6156	0.072*	
H7BC	0.0963	1.0332	0.5639	0.072*	
C8B	0.1591 (3)	0.8972 (2)	0.73143 (17)	0.0510 (7)	
H8BA	0.2066	0.8412	0.7416	0.076*	
H8BB	0.0696	0.8839	0.7366	0.076*	
H8BC	0.1984	0.9475	0.7749	0.076*	
C1C	0.7619 (2)	0.95106 (16)	0.97633 (15)	0.0350 (5)	
C2C	0.7015 (2)	0.89590 (16)	0.89877 (15)	0.0363 (5)	
C3C	0.5886 (3)	0.92509 (19)	0.84673 (16)	0.0458 (6)	
H3C	0.5486	0.8886	0.7951	0.055*	
C4C	0.5352 (2)	1.00708 (18)	0.87041 (16)	0.0434 (6)	
H4C	0.4588	1.0251	0.8349	0.052*	
C5C	0.5937 (2)	1.06415 (17)	0.94707 (16)	0.0395 (6)	
C6C	0.7070 (2)	1.03473 (16)	0.99900 (15)	0.0385 (5)	
H6C	0.7473	1.0718	1.0502	0.046*	
C7C	0.6643 (3)	0.7310 (2)	0.8276 (2)	0.0587 (8)	
H7CA	0.6000	0.7194	0.8637	0.088*	
H7CB	0.7091	0.6742	0.8201	0.088*	
H7CC	0.6218	0.7490	0.7703	0.088*	
C8C	0.8610 (3)	0.8316 (2)	0.8170 (2)	0.0637 (8)	
H8CA	0.8189	0.8569	0.7628	0.096*	
H8CB	0.9013	0.7745	0.8030	0.096*	
H8CC	0.9267	0.8776	0.8497	0.096*	
C1D	0.7193 (2)	0.44478 (18)	−0.02904 (17)	0.0418 (6)	
C2D	0.7337 (2)	0.39226 (18)	−0.10609 (16)	0.0431 (6)	
C3D	0.8142 (3)	0.4285 (2)	−0.15952 (19)	0.0560 (7)	
H3D	0.8221	0.3940	−0.2117	0.067*	
C4D	0.8823 (3)	0.5146 (2)	−0.1365 (2)	0.0570 (7)	
H4D	0.9362	0.5381	−0.1732	0.068*	
C5D	0.8720 (3)	0.56763 (18)	−0.05842 (19)	0.0477 (6)	
C6D	0.7879 (2)	0.53183 (18)	−0.00654 (18)	0.0464 (6)	
H6D	0.7775	0.5673	0.0446	0.056*	
C7D	0.7421 (4)	0.2254 (2)	−0.1671 (2)	0.0863 (12)	
H7DA	0.7604	0.2431	−0.2233	0.129*	
H7DB	0.6941	0.1645	−0.1754	0.129*	
H7DC	0.8230	0.2218	−0.1255	0.129*	
C8D	0.5332 (4)	0.3075 (3)	−0.1930 (3)	0.1163 (18)	
H8DA	0.4854	0.3544	−0.1672	0.174*	
H8DB	0.4833	0.2474	−0.2016	0.174*	
H8DC	0.5484	0.3266	−0.2495	0.174*	

### Atomic displacement parameters (Å^2^)

**Table:**

	*U*^11^	*U*^22^	*U*^33^	*U*^12^	*U*^13^	*U*^23^
S1A	0.0557 (4)	0.0249 (3)	0.0488 (4)	0.0009 (3)	0.0044 (3)	0.0018 (3)
S2A	0.0402 (3)	0.0342 (3)	0.0421 (3)	−0.0021 (2)	0.0077 (3)	−0.0067 (3)
S1B	0.0383 (3)	0.0345 (3)	0.0474 (4)	−0.0011 (2)	0.0069 (3)	−0.0054 (3)
S2B	0.0524 (4)	0.0267 (3)	0.0507 (4)	0.0020 (3)	0.0070 (3)	0.0037 (3)
S1C	0.0371 (3)	0.0453 (4)	0.0428 (4)	−0.0040 (3)	0.0024 (3)	0.0083 (3)
S2C	0.0480 (4)	0.0448 (4)	0.0393 (3)	0.0058 (3)	0.0109 (3)	0.0098 (3)
S1D	0.0471 (4)	0.0546 (4)	0.0591 (4)	0.0077 (3)	0.0228 (3)	0.0164 (3)
S2D	0.0464 (4)	0.0473 (4)	0.0400 (3)	−0.0045 (3)	0.0089 (3)	0.0053 (3)
O1A	0.0602 (12)	0.0481 (11)	0.0633 (12)	−0.0190 (9)	0.0083 (10)	−0.0155 (9)
O2A	0.0684 (13)	0.0674 (13)	0.0461 (11)	−0.0160 (10)	0.0207 (10)	−0.0056 (9)
O3A	0.0599 (12)	0.0586 (12)	0.0607 (12)	0.0230 (10)	−0.0036 (10)	−0.0075 (10)
O1B	0.0626 (13)	0.0545 (12)	0.0621 (12)	0.0253 (10)	−0.0056 (10)	−0.0082 (10)
O2B	0.0588 (12)	0.0774 (14)	0.0526 (12)	−0.0061 (10)	0.0193 (10)	0.0036 (10)
O3B	0.0637 (13)	0.0478 (11)	0.0727 (14)	−0.0182 (9)	0.0085 (11)	−0.0149 (10)
O1C	0.0782 (14)	0.0701 (14)	0.0517 (11)	0.0039 (11)	0.0293 (11)	−0.0032 (10)
O2C	0.0680 (13)	0.0452 (11)	0.0577 (11)	−0.0107 (9)	0.0182 (10)	0.0004 (9)
O3C	0.0591 (13)	0.0903 (16)	0.0842 (15)	0.0214 (12)	0.0141 (12)	0.0499 (13)
O1D	0.0537 (11)	0.0524 (11)	0.0653 (12)	0.0077 (9)	0.0130 (10)	−0.0006 (9)
O2D	0.0725 (14)	0.0822 (15)	0.0527 (12)	−0.0187 (12)	0.0004 (11)	−0.0131 (11)
O3D	0.0548 (12)	0.0899 (16)	0.0757 (14)	0.0013 (11)	0.0221 (11)	0.0434 (12)
N1A	0.0433 (14)	0.0511 (15)	0.0812 (18)	−0.0032 (11)	0.0200 (13)	−0.0013 (13)
N2A	0.0359 (11)	0.0301 (10)	0.0359 (11)	0.0014 (8)	0.0034 (9)	0.0002 (8)
N1B	0.0322 (10)	0.0324 (10)	0.0369 (11)	0.0018 (8)	0.0052 (8)	−0.0008 (8)
N2B	0.0412 (14)	0.0536 (15)	0.0800 (18)	−0.0008 (11)	0.0214 (12)	−0.0008 (13)
N1C	0.0443 (13)	0.0454 (13)	0.0461 (13)	0.0043 (11)	0.0045 (11)	0.0080 (11)
N2C	0.0419 (12)	0.0451 (12)	0.0352 (11)	−0.0018 (9)	0.0101 (9)	−0.0019 (9)
N1D	0.0587 (15)	0.0587 (15)	0.0343 (12)	−0.0145 (11)	0.0063 (11)	0.0082 (10)
N2D	0.0496 (15)	0.0496 (14)	0.0814 (19)	−0.0042 (11)	0.0239 (14)	0.0028 (13)
C1A	0.0384 (13)	0.0280 (11)	0.0312 (11)	0.0032 (9)	0.0031 (10)	0.0035 (9)
C2A	0.0363 (12)	0.0305 (12)	0.0326 (12)	0.0012 (9)	0.0043 (10)	0.0033 (9)
C3A	0.0416 (14)	0.0322 (12)	0.0483 (14)	0.0016 (10)	0.0086 (11)	−0.0039 (11)
C4A	0.0462 (15)	0.0325 (13)	0.0500 (15)	−0.0064 (11)	0.0074 (12)	−0.0032 (11)
C5A	0.0390 (14)	0.0418 (14)	0.0371 (13)	0.0014 (11)	0.0061 (11)	0.0046 (11)
C6A	0.0423 (14)	0.0371 (13)	0.0355 (13)	0.0085 (10)	0.0064 (10)	0.0019 (10)
C7A	0.0530 (17)	0.0684 (19)	0.0510 (16)	0.0038 (14)	0.0162 (14)	0.0205 (14)
C8A	0.0401 (14)	0.0372 (13)	0.0472 (14)	0.0105 (11)	−0.0021 (11)	−0.0038 (11)
C1B	0.0387 (13)	0.0260 (11)	0.0323 (12)	0.0040 (9)	0.0032 (10)	0.0030 (9)
C2B	0.0347 (12)	0.0317 (12)	0.0275 (11)	0.0025 (9)	0.0050 (9)	0.0026 (9)
C3B	0.0408 (13)	0.0288 (12)	0.0361 (12)	0.0047 (10)	0.0084 (10)	0.0012 (10)
C4B	0.0424 (14)	0.0339 (13)	0.0412 (13)	−0.0044 (10)	0.0070 (11)	−0.0010 (10)
C5B	0.0361 (13)	0.0462 (14)	0.0342 (12)	0.0013 (11)	0.0069 (10)	0.0016 (11)
C6B	0.0396 (13)	0.0387 (13)	0.0392 (13)	0.0088 (10)	0.0087 (11)	0.0005 (10)
C7B	0.0368 (14)	0.0405 (14)	0.0628 (17)	0.0099 (11)	0.0019 (12)	−0.0007 (12)
C8B	0.0443 (15)	0.0677 (18)	0.0444 (15)	0.0017 (13)	0.0161 (12)	0.0102 (13)
C1C	0.0358 (12)	0.0369 (13)	0.0313 (12)	−0.0034 (10)	0.0043 (10)	0.0063 (10)
C2C	0.0414 (14)	0.0378 (13)	0.0290 (12)	−0.0053 (10)	0.0082 (10)	0.0011 (10)
C3C	0.0508 (16)	0.0537 (16)	0.0285 (12)	−0.0093 (13)	0.0013 (11)	−0.0008 (11)
C4C	0.0411 (14)	0.0478 (15)	0.0390 (13)	−0.0002 (11)	0.0010 (11)	0.0096 (11)
C5C	0.0420 (14)	0.0389 (13)	0.0389 (13)	−0.0006 (11)	0.0096 (11)	0.0106 (11)
C6C	0.0460 (14)	0.0331 (12)	0.0341 (12)	−0.0049 (10)	0.0042 (11)	0.0029 (10)
C7C	0.0556 (18)	0.0495 (17)	0.0652 (18)	−0.0066 (14)	0.0060 (15)	−0.0155 (14)
C8C	0.073 (2)	0.069 (2)	0.0572 (18)	−0.0051 (16)	0.0384 (16)	−0.0039 (15)
C1D	0.0416 (14)	0.0443 (14)	0.0428 (14)	0.0059 (11)	0.0124 (11)	0.0135 (11)
C2D	0.0471 (15)	0.0427 (14)	0.0391 (13)	−0.0023 (11)	0.0071 (11)	0.0100 (11)
C3D	0.0672 (19)	0.0568 (17)	0.0493 (16)	−0.0014 (14)	0.0253 (14)	0.0048 (13)
C4D	0.0582 (18)	0.0557 (17)	0.0645 (18)	−0.0055 (14)	0.0309 (15)	0.0115 (15)
C5D	0.0434 (15)	0.0433 (15)	0.0601 (17)	0.0048 (12)	0.0161 (13)	0.0120 (13)
C6D	0.0512 (16)	0.0407 (14)	0.0510 (15)	0.0083 (12)	0.0169 (13)	0.0074 (12)
C7D	0.126 (3)	0.060 (2)	0.081 (2)	−0.027 (2)	0.059 (2)	−0.0250 (18)
C8D	0.095 (3)	0.128 (4)	0.097 (3)	−0.051 (3)	−0.052 (2)	0.057 (3)

### Geometric parameters (Å, °)

**Table:**

S1A—C1A	1.775 (2)	C4A—H4A	0.9300
S1A—S2A	2.1105 (9)	C5A—C6A	1.388 (3)
S2A—O2A	1.4342 (19)	C6A—H6A	0.9300
S2A—O3A	1.4346 (19)	C7A—H7AA	0.9600
S2A—O1A	1.4411 (18)	C7A—H7AB	0.9600
S1B—O2B	1.4328 (19)	C7A—H7AC	0.9600
S1B—O1B	1.4339 (19)	C8A—H8AA	0.9600
S1B—O3B	1.4411 (18)	C8A—H8AB	0.9600
S1B—S2B	2.1194 (9)	C8A—H8AC	0.9600
S2B—C1B	1.777 (2)	C1B—C6B	1.383 (3)
S1C—C1C	1.769 (2)	C1B—C2B	1.397 (3)
S1C—S2C	2.1267 (9)	C2B—C3B	1.384 (3)
S2C—O3C	1.428 (2)	C3B—C4B	1.376 (3)
S2C—O1C	1.429 (2)	C3B—H3B	0.9300
S2C—O2C	1.441 (2)	C4B—C5B	1.402 (3)
S1D—C1D	1.775 (2)	C4B—H4B	0.9300
S1D—S2D	2.1233 (10)	C5B—C6B	1.393 (3)
S2D—O2D	1.428 (2)	C6B—H6B	0.9300
S2D—O3D	1.4376 (19)	C7B—H7BA	0.9600
S2D—O1D	1.4396 (19)	C7B—H7BB	0.9600
N1A—C5A	1.367 (3)	C7B—H7BC	0.9600
N1A—H1AA	0.86 (3)	C8B—H8BA	0.9600
N1A—H1AB	0.89 (3)	C8B—H8BB	0.9600
N2A—C2A	1.470 (3)	C8B—H8BC	0.9600
N2A—C7A	1.491 (3)	C1C—C6C	1.393 (3)
N2A—C8A	1.492 (3)	C1C—C2C	1.400 (3)
N2A—H2A	0.81 (3)	C2C—C3C	1.385 (3)
N1B—C2B	1.473 (3)	C3C—C4C	1.369 (4)
N1B—C7B	1.492 (3)	C3C—H3C	0.9300
N1B—C8B	1.492 (3)	C4C—C5C	1.398 (3)
N1B—H1B	0.86 (2)	C4C—H4C	0.9300
N2B—C5B	1.363 (3)	C5C—C6C	1.388 (3)
N2B—H2BA	0.86 (2)	C6C—H6C	0.9300
N2B—H2BB	0.87 (3)	C7C—H7CA	0.9600
N1C—C5C	1.379 (3)	C7C—H7CB	0.9600
N1C—H1CA	0.81 (3)	C7C—H7CC	0.9600
N1C—H1CB	0.83 (3)	C8C—H8CA	0.9600
N2C—C2C	1.468 (3)	C8C—H8CB	0.9600
N2C—C8C	1.496 (3)	C8C—H8CC	0.9600
N2C—C7C	1.497 (3)	C1D—C6D	1.379 (3)
N2C—H2C	0.82 (3)	C1D—C2D	1.392 (3)
N1D—C2D	1.479 (3)	C2D—C3D	1.380 (3)
N1D—C8D	1.500 (4)	C3D—C4D	1.366 (4)
N1D—C7D	1.500 (4)	C3D—H3D	0.9300
N1D—H1D	0.82 (3)	C4D—C5D	1.396 (4)
N2D—C5D	1.392 (4)	C4D—H4D	0.9300
N2D—H2DA	0.79 (3)	C5D—C6D	1.387 (3)
N2D—H2DB	0.99 (3)	C6D—H6D	0.9300
C1A—C6A	1.383 (3)	C7D—H7DA	0.9600
C1A—C2A	1.396 (3)	C7D—H7DB	0.9600
C2A—C3A	1.389 (3)	C7D—H7DC	0.9600
C3A—C4A	1.370 (3)	C8D—H8DA	0.9600
C3A—H3A	0.9300	C8D—H8DB	0.9600
C4A—C5A	1.399 (3)	C8D—H8DC	0.9600
			
C1A—S1A—S2A	100.38 (8)	H8AA—C8A—H8AC	109.5
O2A—S2A—O3A	113.54 (13)	H8AB—C8A—H8AC	109.5
O2A—S2A—O1A	113.29 (11)	C6B—C1B—C2B	119.4 (2)
O3A—S2A—O1A	115.94 (12)	C6B—C1B—S2B	119.89 (17)
O2A—S2A—S1A	106.64 (9)	C2B—C1B—S2B	120.70 (17)
O3A—S2A—S1A	104.90 (9)	C3B—C2B—C1B	119.8 (2)
O1A—S2A—S1A	100.82 (9)	C3B—C2B—N1B	120.32 (19)
O2B—S1B—O1B	113.64 (13)	C1B—C2B—N1B	119.88 (19)
O2B—S1B—O3B	113.81 (12)	C4B—C3B—C2B	120.2 (2)
O1B—S1B—O3B	115.59 (13)	C4B—C3B—H3B	119.9
O2B—S1B—S2B	106.63 (9)	C2B—C3B—H3B	119.9
O1B—S1B—S2B	104.64 (9)	C3B—C4B—C5B	121.4 (2)
O3B—S1B—S2B	100.73 (9)	C3B—C4B—H4B	119.3
C1B—S2B—S1B	99.21 (8)	C5B—C4B—H4B	119.3
C1C—S1C—S2C	99.12 (8)	N2B—C5B—C6B	121.8 (2)
O3C—S2C—O1C	116.76 (14)	N2B—C5B—C4B	120.6 (2)
O3C—S2C—O2C	113.72 (14)	C6B—C5B—C4B	117.5 (2)
O1C—S2C—O2C	111.87 (13)	C1B—C6B—C5B	121.7 (2)
O3C—S2C—S1C	100.41 (9)	C1B—C6B—H6B	119.1
O1C—S2C—S1C	107.73 (9)	C5B—C6B—H6B	119.1
O2C—S2C—S1C	104.76 (8)	N1B—C7B—H7BA	109.5
C1D—S1D—S2D	99.41 (9)	N1B—C7B—H7BB	109.5
O2D—S2D—O3D	115.78 (14)	H7BA—C7B—H7BB	109.5
O2D—S2D—O1D	112.56 (12)	N1B—C7B—H7BC	109.5
O3D—S2D—O1D	114.26 (13)	H7BA—C7B—H7BC	109.5
O2D—S2D—S1D	107.46 (11)	H7BB—C7B—H7BC	109.5
O3D—S2D—S1D	100.31 (9)	N1B—C8B—H8BA	109.5
O1D—S2D—S1D	104.81 (9)	N1B—C8B—H8BB	109.5
C5A—N1A—H1AA	119 (2)	H8BA—C8B—H8BB	109.5
C5A—N1A—H1AB	122 (2)	N1B—C8B—H8BC	109.5
H1AA—N1A—H1AB	116 (3)	H8BA—C8B—H8BC	109.5
C2A—N2A—C7A	111.71 (19)	H8BB—C8B—H8BC	109.5
C2A—N2A—C8A	113.43 (18)	C6C—C1C—C2C	118.9 (2)
C7A—N2A—C8A	111.0 (2)	C6C—C1C—S1C	119.49 (18)
C2A—N2A—H2A	108.8 (19)	C2C—C1C—S1C	121.57 (18)
C7A—N2A—H2A	109.6 (19)	C3C—C2C—C1C	119.6 (2)
C8A—N2A—H2A	101.8 (19)	C3C—C2C—N2C	121.1 (2)
C2B—N1B—C7B	113.76 (18)	C1C—C2C—N2C	119.3 (2)
C2B—N1B—C8B	111.14 (18)	C4C—C3C—C2C	120.8 (2)
C7B—N1B—C8B	111.0 (2)	C4C—C3C—H3C	119.6
C2B—N1B—H1B	108.5 (17)	C2C—C3C—H3C	119.6
C7B—N1B—H1B	105.6 (17)	C3C—C4C—C5C	121.1 (2)
C8B—N1B—H1B	106.4 (17)	C3C—C4C—H4C	119.5
C5B—N2B—H2BA	120 (2)	C5C—C4C—H4C	119.5
C5B—N2B—H2BB	126 (2)	N1C—C5C—C6C	121.3 (2)
H2BA—N2B—H2BB	114 (3)	N1C—C5C—C4C	120.6 (2)
C5C—N1C—H1CA	116 (2)	C6C—C5C—C4C	118.0 (2)
C5C—N1C—H1CB	112 (2)	C5C—C6C—C1C	121.7 (2)
H1CA—N1C—H1CB	117 (3)	C5C—C6C—H6C	119.2
C2C—N2C—C8C	111.0 (2)	C1C—C6C—H6C	119.2
C2C—N2C—C7C	114.1 (2)	N2C—C7C—H7CA	109.5
C8C—N2C—C7C	111.1 (2)	N2C—C7C—H7CB	109.5
C2C—N2C—H2C	109.4 (19)	H7CA—C7C—H7CB	109.5
C8C—N2C—H2C	105 (2)	N2C—C7C—H7CC	109.5
C7C—N2C—H2C	105 (2)	H7CA—C7C—H7CC	109.5
C2D—N1D—C8D	111.1 (2)	H7CB—C7C—H7CC	109.5
C2D—N1D—C7D	113.7 (2)	N2C—C8C—H8CA	109.5
C8D—N1D—C7D	112.6 (3)	N2C—C8C—H8CB	109.5
C2D—N1D—H1D	110 (2)	H8CA—C8C—H8CB	109.5
C8D—N1D—H1D	103 (2)	N2C—C8C—H8CC	109.5
C7D—N1D—H1D	106 (2)	H8CA—C8C—H8CC	109.5
C5D—N2D—H2DA	118 (2)	H8CB—C8C—H8CC	109.5
C5D—N2D—H2DB	114.4 (18)	C6D—C1D—C2D	119.2 (2)
H2DA—N2D—H2DB	111 (3)	C6D—C1D—S1D	119.8 (2)
C6A—C1A—C2A	119.5 (2)	C2D—C1D—S1D	120.95 (19)
C6A—C1A—S1A	119.55 (17)	C3D—C2D—C1D	119.8 (2)
C2A—C1A—S1A	120.89 (17)	C3D—C2D—N1D	119.8 (2)
C3A—C2A—C1A	119.5 (2)	C1D—C2D—N1D	120.4 (2)
C3A—C2A—N2A	120.5 (2)	C4D—C3D—C2D	120.6 (3)
C1A—C2A—N2A	120.00 (19)	C4D—C3D—H3D	119.7
C4A—C3A—C2A	120.1 (2)	C2D—C3D—H3D	119.7
C4A—C3A—H3A	120.0	C3D—C4D—C5D	120.7 (2)
C2A—C3A—H3A	120.0	C3D—C4D—H4D	119.7
C3A—C4A—C5A	121.6 (2)	C5D—C4D—H4D	119.7
C3A—C4A—H4A	119.2	C6D—C5D—N2D	120.2 (3)
C5A—C4A—H4A	119.2	C6D—C5D—C4D	118.2 (2)
N1A—C5A—C6A	121.7 (2)	N2D—C5D—C4D	121.4 (2)
N1A—C5A—C4A	120.7 (2)	C1D—C6D—C5D	121.5 (2)
C6A—C5A—C4A	117.6 (2)	C1D—C6D—H6D	119.3
C1A—C6A—C5A	121.7 (2)	C5D—C6D—H6D	119.3
C1A—C6A—H6A	119.2	N1D—C7D—H7DA	109.5
C5A—C6A—H6A	119.2	N1D—C7D—H7DB	109.5
N2A—C7A—H7AA	109.5	H7DA—C7D—H7DB	109.5
N2A—C7A—H7AB	109.5	N1D—C7D—H7DC	109.5
H7AA—C7A—H7AB	109.5	H7DA—C7D—H7DC	109.5
N2A—C7A—H7AC	109.5	H7DB—C7D—H7DC	109.5
H7AA—C7A—H7AC	109.5	N1D—C8D—H8DA	109.5
H7AB—C7A—H7AC	109.5	N1D—C8D—H8DB	109.5
N2A—C8A—H8AA	109.5	H8DA—C8D—H8DB	109.5
N2A—C8A—H8AB	109.5	N1D—C8D—H8DC	109.5
H8AA—C8A—H8AB	109.5	H8DA—C8D—H8DC	109.5
N2A—C8A—H8AC	109.5	H8DB—C8D—H8DC	109.5
			
C1A—S1A—S2A—O2A	62.82 (12)	C3B—C4B—C5B—N2B	−176.6 (2)
C1A—S1A—S2A—O3A	−57.91 (12)	C3B—C4B—C5B—C6B	1.7 (3)
C1A—S1A—S2A—O1A	−178.68 (11)	C2B—C1B—C6B—C5B	−0.7 (3)
O2B—S1B—S2B—C1B	61.78 (12)	S2B—C1B—C6B—C5B	178.81 (18)
O1B—S1B—S2B—C1B	−58.93 (12)	N2B—C5B—C6B—C1B	177.6 (2)
O3B—S1B—S2B—C1B	−179.18 (12)	C4B—C5B—C6B—C1B	−0.6 (4)
C1C—S1C—S2C—O3C	165.76 (14)	S2C—S1C—C1C—C6C	98.42 (18)
C1C—S1C—S2C—O1C	−71.61 (13)	S2C—S1C—C1C—C2C	−81.58 (19)
C1C—S1C—S2C—O2C	47.64 (12)	C6C—C1C—C2C—C3C	−0.4 (3)
C1D—S1D—S2D—O2D	70.29 (13)	S1C—C1C—C2C—C3C	179.58 (18)
C1D—S1D—S2D—O3D	−168.34 (13)	C6C—C1C—C2C—N2C	177.8 (2)
C1D—S1D—S2D—O1D	−49.66 (13)	S1C—C1C—C2C—N2C	−2.2 (3)
S2A—S1A—C1A—C6A	−99.70 (18)	C8C—N2C—C2C—C3C	91.1 (3)
S2A—S1A—C1A—C2A	83.71 (18)	C7C—N2C—C2C—C3C	−35.3 (3)
C6A—C1A—C2A—C3A	−0.7 (3)	C8C—N2C—C2C—C1C	−87.1 (3)
S1A—C1A—C2A—C3A	175.85 (17)	C7C—N2C—C2C—C1C	146.5 (2)
C6A—C1A—C2A—N2A	−179.5 (2)	C1C—C2C—C3C—C4C	−0.2 (4)
S1A—C1A—C2A—N2A	−2.9 (3)	N2C—C2C—C3C—C4C	−178.4 (2)
C7A—N2A—C2A—C3A	−87.4 (3)	C2C—C3C—C4C—C5C	0.7 (4)
C8A—N2A—C2A—C3A	39.0 (3)	C3C—C4C—C5C—N1C	176.2 (2)
C7A—N2A—C2A—C1A	91.4 (3)	C3C—C4C—C5C—C6C	−0.6 (3)
C8A—N2A—C2A—C1A	−142.3 (2)	N1C—C5C—C6C—C1C	−176.8 (2)
C1A—C2A—C3A—C4A	0.9 (4)	C4C—C5C—C6C—C1C	0.0 (3)
N2A—C2A—C3A—C4A	179.7 (2)	C2C—C1C—C6C—C5C	0.5 (3)
C2A—C3A—C4A—C5A	−0.5 (4)	S1C—C1C—C6C—C5C	−179.46 (17)
C3A—C4A—C5A—N1A	−178.3 (3)	S2D—S1D—C1D—C6D	−93.5 (2)
C3A—C4A—C5A—C6A	0.0 (4)	S2D—S1D—C1D—C2D	88.2 (2)
C2A—C1A—C6A—C5A	0.2 (3)	C6D—C1D—C2D—C3D	−1.1 (4)
S1A—C1A—C6A—C5A	−176.44 (18)	S1D—C1D—C2D—C3D	177.2 (2)
N1A—C5A—C6A—C1A	178.4 (2)	C6D—C1D—C2D—N1D	179.6 (2)
C4A—C5A—C6A—C1A	0.2 (4)	S1D—C1D—C2D—N1D	−2.1 (3)
S1B—S2B—C1B—C6B	−99.62 (19)	C8D—N1D—C2D—C3D	−87.0 (4)
S1B—S2B—C1B—C2B	79.84 (18)	C7D—N1D—C2D—C3D	41.2 (4)
C6B—C1B—C2B—C3B	0.9 (3)	C8D—N1D—C2D—C1D	92.3 (3)
S2B—C1B—C2B—C3B	−178.55 (17)	C7D—N1D—C2D—C1D	−139.5 (3)
C6B—C1B—C2B—N1B	−176.6 (2)	C1D—C2D—C3D—C4D	1.5 (4)
S2B—C1B—C2B—N1B	3.9 (3)	N1D—C2D—C3D—C4D	−179.2 (3)
C7B—N1B—C2B—C3B	34.7 (3)	C2D—C3D—C4D—C5D	0.0 (5)
C8B—N1B—C2B—C3B	−91.5 (2)	C3D—C4D—C5D—C6D	−1.9 (4)
C7B—N1B—C2B—C1B	−147.8 (2)	C3D—C4D—C5D—N2D	−177.6 (3)
C8B—N1B—C2B—C1B	86.0 (3)	C2D—C1D—C6D—C5D	−0.7 (4)
C1B—C2B—C3B—C4B	0.2 (3)	S1D—C1D—C6D—C5D	−179.1 (2)
N1B—C2B—C3B—C4B	177.7 (2)	N2D—C5D—C6D—C1D	178.0 (3)
C2B—C3B—C4B—C5B	−1.5 (4)	C4D—C5D—C6D—C1D	2.2 (4)

### Hydrogen-bond geometry (Å, °)

**Table:**

*D*—H···*A*	*D*—H	H···*A*	*D*···*A*	*D*—H···*A*
N1A—H1AA···O2A^i^	0.86 (3)	2.40 (3)	3.204 (3)	157 (3)
N1A—H1AB···O3B^i^	0.89 (3)	2.38 (3)	3.189 (3)	152 (3)
N2A—H2A···O3A	0.81 (3)	2.31 (2)	2.983 (3)	141 (2)
N2A—H2A···O3B^ii^	0.81 (2)	2.48 (2)	3.003 (3)	124 (3)
N1B—H1B···O1B	0.86 (2)	2.28 (3)	2.940 (3)	134 (2)
N1B—H1B···O1A^ii^	0.86 (2)	2.45 (2)	3.016 (3)	124 (1)
N2B—H2BA···O2B^iii^	0.86 (2)	2.48 (2)	3.266 (3)	153 (3)
N2B—H2BB···O1A^i^	0.87 (3)	2.39 (3)	3.237 (3)	163 (3)
N1C—H1CA···O2C^iv^	0.81 (3)	2.44 (3)	3.149 (3)	147 (3)
N1C—H1CB···O3D^v^	0.83 (3)	2.55 (3)	3.349 (3)	162 (3)
N2C—H2C···O2C	0.82 (3)	2.41 (3)	2.982 (3)	128 (3)
N2C—H2C···N2D^vi^	0.82 (2)	2.44 (3)	3.122 (4)	143 (3)
N1D—H1D···N1C^vii^	0.81 (3)	2.31 (3)	3.013 (3)	146 (3)
N2D—H2DA···O1D^viii^	0.79 (3)	2.30 (3)	3.040 (4)	155 (3)
N2D—H2DB···O3C^ix^	0.99 (3)	2.40 (3)	3.314 (4)	154 (2)
C8A—H8AA···O2D^i^	0.96	2.37	3.273 (3)	157
C8B—H8BA···O3D^i^	0.96	2.54	3.437 (4)	155
C7D—H7DA···O2B^x^	0.96	2.51	3.370 (4)	149

Symmetry codes: (i) −*x*+1, −*y*+1, −*z*+1; (ii) −*x*, −*y*+1, −*z*+1; (iii) −*x*+1, −*y*+2, −*z*+1; (iv) −*x*+1, −*y*+2, −*z*+2; (v) *x*, *y*+1, *z*+1; (vi) *x*, *y*, *z*+1; (vii) *x*, *y*−1, *z*−1; (viii) −*x*+2, −*y*+1, −*z*; (ix) *x*, *y*, *z*−1; (x) −*x*+1, −*y*+1, −*z*.
